# High-throughput protein production and purification at the Seattle Structural Genomics Center for Infectious Disease

**DOI:** 10.1107/S1744309111018367

**Published:** 2011-08-13

**Authors:** Cassie M. Bryan, Janhavi Bhandari, Alberto J. Napuli, David J. Leibly, Ryan Choi, Angela Kelley, Wesley C. Van Voorhis, Thomas E. Edwards, Lance J. Stewart

**Affiliations:** a Seattle Structural Genomics Center for Infectious Disease (SSGCID), USA; b Division of Allergy and Infectious Diseases, School of Medicine, University of Washington, MS 356423, Seattle, WA 98195-6423, USA; c Emerald BioStructures Inc., 7869 NE Day Road West, Bainbridge Island, WA 98110, USA

**Keywords:** protein production, purification, immobilized metal-affinity chromatography, size-exclusion chromatography, structural genomics, 3C protease, enzymatic cleavage

## Abstract

The establishment of an efficient and reliable protein-purification pipeline is essential for the success of structural genomic projects. The SSGCID Protein Purification Group at the University of Washington (UW-PPG) has established a robust protein-purification pipeline designed to purify 400 proteins per year at a rate of eight purifications per week. The pipeline was implemented using two ÄKTAexplorer 100s and four ÄKTAprimes to perform immobilized metal-affinity chromatography (IMAC) and size-exclusion chromatography. Purifications were completed in a period of 5 d and yielded an average of 53 mg highly purified protein. This paper provides a detailed description of the methods used to purify, characterize and store SSGCID proteins. Some of the purified proteins were treated with 3C protease, which was expressed and purified by UW-PPG using a similar protocol, to cleave non-native six-histidine tags. The cleavage was successful in 94% of 214 attempts. Cleaved proteins yielded 2.9% more structures than uncleaved six-histidine-tagged proteins. This 2.9% improvement may seem small, but over the course of the project the structure output from UW-PPG is thus predicted to increase from 260 structures to 318 structures. Therefore, the outlined protocol with 3C cleavage and subtractive IMAC has been shown to be a highly efficient method for the standardized purification of recombinant proteins for structure determination *via* X-ray crystallography.

## Abstract 2

An overview of the standard SSGCID protein-purification protocol is given and success rates and cleavage alternatives are discussed.

## 1. Introduction

**Figure 1:**
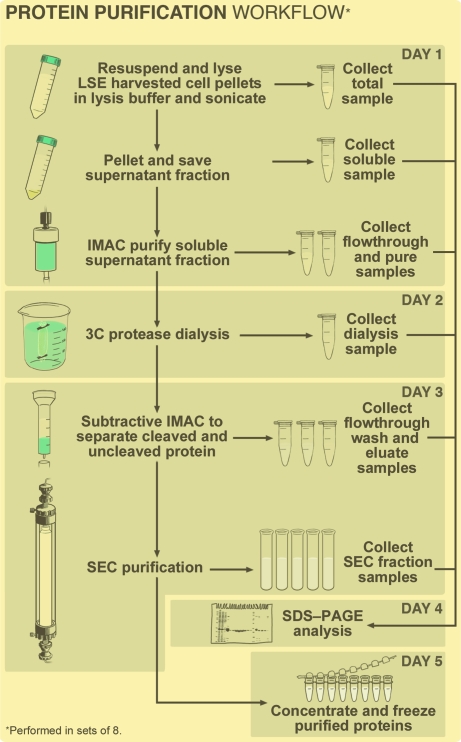
Flowchart of the UW-PPG protein-purification protocol. Eight SSGCID targets were purified per week utilizing two research scientists, two ÄTKAexplorer 100s and four ÄTKAprimes (GE Healthcare, Piscataway, New Jersey, USA). Following initial immobilized metal-affinity chromatography (IMAC) of the soluble lysates, the polyhistidine tag was removed from the recombinant protein using 3C protease. The cleaved protein was separated from the 3C protease, the His-tag peptide, uncleaved protein and any Ni-binding contaminants through subtractive IMAC. Size-exclusion chromatography (SEC) was then used as a final purification step and SDS–PAGE was used to determine the fractions to pool. The pooled protein was concentrated to 20–30 mg ml^−1^ and stored at 193 K. In our group, the procedures were carried out on the days noted in the upper right-hand corner of each box.

The Seattle Structural Genomics Center for Infectious Disease (SSGCID) was established as a collaboration between Seattle BioMed, Emerald BioSystems and the University of Washington in 2007. Its aim is to solve three-dimensional structures of pathogenic proteins from various organisms listed as category A–C agents according to the National Institute of Allergy and Infectious Diseases (NIAID) at a rate of 75–100 per year. Owing to the intensity of this goal, the implementation of a robust protein-purification pipeline was an essential requirement for the success of the SSGCID project. The primary objective was to develop a standard operating procedure (SOP) that would support the purification of 400 crystal-quality proteins per year at a rate of eight purifications per week (Fig. 1 ▶). To accomplish this task, the Protein Purification Group at the University of Washington (UW-PPG) employed two full-time research scientists, two ÄTKAexplorer 100s and four ÄTKAprimes (GE Healthcare, Piscataway, New Jersey, USA). One of the key criteria in the design of the UW-PPG protein-purification strategy was that the weekly goals had to be completed in a period of five working days in order to fit within the laboratory schedule. Following this approach, the UW-PPG implemented a semi-automated protein-purification pipeline based on the capture of bacterial and eukaryotic proteins with N-­terminal histidine tags using metal-affinity chromatography followed by the cleavage of the N-terminal 6-His tags with 6-His-MBP-3C protease, which is expressed and purified in-house, and size-exclusion chromatography (SEC). This paper presents a detailed description of the UW-PPG protein-purification SOP and, most importantly, discusses success rates to demonstrate the efficiency of the outlined protocol.

## 2. Materials and methods

### 2.1. Cloning and expression testing

Open reading frames encoding the selected protein targets were PCR-amplified in a 96-well format using either genomic DNA or cDNA as a template. The PCR primers were designed with an additional ligation-independent cloning (LIC) sequence at their 5′ ends that was complementary to the LIC sequence in the plasmid vector (Choi *et al.*, 2011 ▶). Purified PCR products were then cloned *via* LIC (Aslanidis & de Jong, 1990 ▶) into the AVA0421 expression vector (received as a gift from Dr Elizabeth Grayhack; Quartley *et al.*, 2009 ▶), which was derived from pET14b and provides cleavable six-histidine tags (His tags) at the N-termini of the expressed proteins (the 3C protease recognition sequence is Leu-Glu-Ala-Gln-Thr-Gln*-Gly-Pro, where * is the cleavage site; Alexandrov *et al.*, 2004 ▶; Choi *et al.*, 2011 ▶). The recombinant plasmids were transformed into *Escherichia coli* Rosetta Oxford strain [BL21*(DE3)-R3-pRARE2] cells for expression testing (Choi *et al.*, 2011 ▶) and the proteins which showed solubility continued to large-scale expression.

Inoculum cultures of lysogeny broth (LB) with appropriate antibiotics were grown for approximately 16 h at 310 K as described by Choi *et al.* (2011 ▶). ZYP-5052 auto-induction medium was freshly prepared as per Studier’s published protocol (Studier, 2005 ▶). Antibiotics (50 µg ml^−1^ ampicillin, 50 µg ml^−1^ carbenicillin and/or 34 µg ml^−1^ chloramphenicol, depending on strain/plasmid concentration) were added to Pyrex bottles containing 2 l sterile auto-induction medium as well as 400 µl antifoam (Sigma, St Louis, USA; Choi *et al.*, 2011 ▶). The bottles were inoculated with 3 ml overnight culture and placed into a LEX bioreactor (Harbinger Biotech, Ontario, Canada). The cultures were grown for approximately 24 h at 298 K; the temperature was then dropped to 288 K for approximately 72 h. To harvest, the culture was centrifuged at 4000*g* for 20 min at 277 K. The cell paste was flash-frozen in liquid nitrogen and stored at 193 K. Large-scale expressions were qualitatively analyzed by performing a high-throughput screen to determine the level of expression and solubility prior to purification (Choi *et al.*, 2011 ▶).

### 2.2. Protein purification

Frozen bacterial cell pellets (averaging 25 g) were resuspended in lysis buffer (25 m*M* HEPES, 500 m*M* NaCl, 5% glycerol, 30 m*M* imidazole, 0.025% sodium azide, 0.5% CHAPS, 10 m*M* MgCl_2_, 1 m*M* TCEP, 250 µg ml^−1^ AEBSF, 0.05  µg ml^−1^ lysozyme pH 7.0). Cells underwent sonication on ice using a Virtis Versonic 600 sonicator (SP Scientific, Gardiner, New York, USA) programmed to run for 30 min in 15 s intervals at 100 W separated by 15 s resting time. The cell debris was incubated with 20 µl Benzonase nuclease (25 units ml^−1^; EMD Chemicals, San Diego, California, USA) at room temperature for 45 min and a ‘total’ sample was taken for subsequent analysis by SDS–PAGE. Clarification was achieved by centrifugation at 29 774*g* for 75 min at 277 K and a ‘soluble’ sample was collected. Immobilized metal-affinity chromatography (IMAC) removed the majority of the native *E. coli* proteins using HisTrap FF 5 ml columns (GE Healthcare, Piscataway, New Jersey, USA) equilibrated with wash buffer (25 m*M* HEPES, 500 m*M* NaCl, 5% glycerol, 30 m*M* imidazole, 0.025% sodium azide, 1 m*M* TCEP pH 7.0). The soluble lysate was loaded using an ÄKTAexplorer 100 (GE Healthcare, Piscataway, New York, USA). The flowthrough was collected and a sample was saved. 20 column volumes of wash buffer were run over the column to remove any unbound protein. The His-tagged protein and any other Ni-binding proteins (Bolanos-Garcia & Davies, 2006 ▶) were eluted with seven column volumes of elution buffer (25 m*M* HEPES, 500 m*M* NaCl, 5% glycerol, 1 m*M* TCEP, 250 m*M* imidazole and 0.025% azide pH 7.0) and collected in 3 ml fractions. The OD_280_ absorbance chromatogram was used to determine which fractions to pool.

Cleavage of the His tag from the target protein was achieved by ‘in-­solution’ digestion in the presence of 3C protease. However, it is important to note that single-step ‘on-column’ cleavage and separation of the tagless protein from 3C protease has also been reported to be successful (Hedhammar *et al.*, 2006 ▶). The advantages of the ‘in-solution’ technique are that multiple samples can be run in parallel; the proteins are freely diffusible so that constraints of the protease needing to be adjacent to a protein are not operant and the cleavage can proceed further to completion. Owing to the high-throughput nature of the SSGCID project, the ability to fully cleave four proteins simultaneously outweighs the extra time spent performing a separate subtractive IMAC step, making the ‘in-solution’ method more practical in this case. 3C protease was added to the protein at a ratio of 1:50(*w*:*w*) and the mixture was dialyzed overnight (generally 18 h) at 277 K in dialysis buffer (25 m*M* HEPES, 500 m*M* NaCl, 5% glycerol, 1 m*M* TCEP and 0.025% azide pH 7.5).

A second IMAC step was used to remove uncleaved protein, the His-tag peptide, any Ni-binding *E. coli* contaminant proteins and the His-tagged 3C protease from the cleaved protein. The sample was loaded onto a gravity-flow column (Econo-Pac Chromatography Columns, Bio-Rad, Hercules, California, USA) packed with pre-equilibrated Ni Sepharose (2.5 or 5 ml depending on the protein yield; GE Healthcare, Piscataway, New Jersey) and the flowthrough was collected. Two column volumes of wash buffer (the same as for the first IMAC) purged the resin of unbound sample and this wash fraction was also collected. The Ni-bound proteins (ideally, 3C protease, non-His-tagged protein contaminants and uncleaved protein) were collected from the column upon the addition of four column volumes of elution buffer (also the same as for the first IMAC). Qualitative analysis of the digestion reaction was performed by SDS–PAGE and quantitative analysis was performed by measuring the concentration of protein in the flowthrough, wash and eluate samples. After determining where the target protein eluted, the appropriate fraction(s) were concentrated (Amicon Ultra-15 Centrifugal Filter Units, Millipore, Carrigtwohill, Ireland) to approximately 10–15 ml in preparation for size-exclusion chromatography (SEC).

Purification was completed by performing SEC as a final step. The cleaved protein was loaded using an ÄKTAexplorer or ÄKTAprime (GE Healthcare, Piscataway, New Jersey, USA) onto a HiLoad 26/60 Superdex 75 preparative-grade column (GE Healthcare, Piscataway, New Jersey, USA) that had previously been equilibrated in SEC buffer (25 m*M* HEPES, 500 m*M* NaCl, 5% glycerol, 2 m*M* DTT, 0.025% azide pH 7.0) and the eluate was collected in 5 ml fractions. The apparent molecular weight of the eluted protein was determined based on the elution volume and a standard calibration curve for the column to give an estimate of the oligomeric state of the protein. SEC fractions and in-process samples were analyzed by SDS–PAGE to confirm the success of purification and determine which SEC fractions to pool for final concentration. After pooling the appropriate SEC fractions, the protein was concentrated using an Amicon Ultra-15 Centrifugal Filter Unit (Millipore, Carrigtwohill, Ireland) to 20–30 mg ml^−1^. 100–200 µl aliquots were then flash-frozen in flexible eight-well strips (PCR strip tubes, Axygen, Union City, California, USA) using liquid nitrogen and stored at 193 K.

### 2.3. Production of 6-His-MBP-3C protease

An engineered form of 3C protease was used for the removal of non-native histidine tags from the N-terminus of recombinant target proteins. This 6-His-MBP-3C protease construct was a generous gift from Professor Eric Pfiziky of the University of Rochester. It was expressed following the same protocol as used for the large-scale expression of SSGCID target proteins. 6-His-MBP-3C protease was purified in three steps including primary IMAC, SEC and dialysis into storage buffer. IMAC and SEC were performed just as they were for other SSGCID proteins, with all of the buffers remaining the same except for the lysis buffer, which did not contain the protease inhibitor AEBSF (25 m*M* HEPES, 500 m*M* NaCl, 5% glycerol, 30 m*M* imidazole, 0.025% sodium azide, 0.5% CHAPS, 10 m*M* MgCl_2_, 1 m*M* TCEP, 0.05 µg ml^−1^ lysozyme pH 7.0). Following SEC, peak fractions were confirmed by SDS–PAGE analysis, pooled and concentrated to 6–7 mg ml^−1^. The concentrated sample was then dialyzed overnight into storage buffer (25 m*M *HEPES, 200 m*M* NaCl, 1 m*M* TCEP, 50% glycerol pH 7.5). During dialysis, the concentration of 6-His-MBP-3C protease generally increased to 12–20 mg ml^−1^ owing to the much higher glycerol concentration in the storage buffer (50% glycerol) *versus* the SEC buffer (5% glycerol) going into dialysis. The purified 6-His-MBP-3C protease was then stored at 253 K.

## 3. Results and discussion

**Figure 2:**
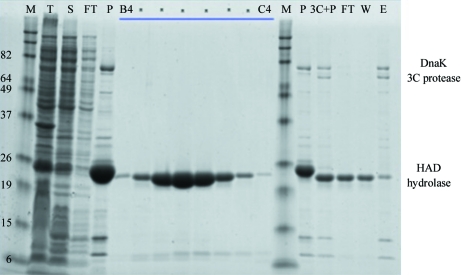
A GelCode Blue-stained (Thermo Scientific, Rockford, Illinois, USA) SDS–PAGE of samples from a typical purification, represented in this case by recombinant HAD-superfamily hydrolase from *Ehrlichia chaffeensis*. Lanes are labelled as follows: M, molecular-weight standards; T, total protein; S, soluble fraction; FT, flowthrough (nonbound) from the first IMAC column; P, purified protein after first IMAC column; B4–C4, successive size-exclusion chromatography (SEC) fractions from peak (see Fig. 2 ▶), the dotted fractions were pooled for final concentration; 3C+P, protein after overnight cleavage with 3C protease; FT, unbound protein from second IMAC column after dialysis with 3C protease; W, protein from second IMAC column that eluted in the wash fractions; E, protein eluted from the second IMAC column with 500 m*M* imidazole. The identity of the DnaK protein band was determined by gel extraction, trypsin digest and mass-spectrometric analysis.

An example of a typical purification is that of the 24 kDa HAD-superfamily hydrolase found in *Ehrlichia chaffeensis* (PDB entry 3kzx). The large-scale culture yielded medium expression levels with medium solubility, as demonstrated by the total (T) and soluble (S) lanes on the SDS–PAGE image (Fig. 2 ▶). The first IMAC was successful in removing most of the *E. coli* background proteins [flowthrough (FT) and pure (P) lanes on the left-hand side of the SDS–PAGE] and 76 mg total protein was recovered. 3C protease successfully cleaved all of the protein and a visible shift of about 2 kDa was seen on the gel. 68% of the protein (52 mg) was recovered in the flowthrough (FT; right-hand side of SDS–PAGE) and wash (W) portions of the subtractive IMAC step, while the elution (E) portion contained the rest of the cleaved target protein, the 3C protease and DnaK, a metal-binding heat-shock protein native to *E. coli* (Baneyx & Nannenga, 2010 ▶). SEC was run on the flowthrough and wash fractions and yielded a single symmetrical peak (fractions B4–C4 containing the peak are seen in Fig. 2 ▶). After pooling the appropriate fractions (pooled fractions are marked in Fig. 2 ▶), the purified protein was concentrated to 1.6 ml at 26.8 mg ml^−1^ and stored at 193 K.

Including enzymatic cleavage and subtractive IMAC in our standard protocol not only removes the non-native His tag but also generally improves the purity of the protein. 3C protease was used for enzymatic cleavage as it is active at 277 K in a wide range of buffers (Tris, imidazole, PBS) including salt concentrations of 0.1–0.5 *M* with a pH range of 6.8–8.2 (Walker *et al.*, 1994 ▶). While no data have been collected showing that maltose-binding protein (MBP) increases the expression or solubility of 3C protease, MBP-fusion data have been published for TEV protease, another protease that is very commonly used by structural genomic centers. In one case, the use of an MBP fusion, together with other factors such as the use of an autoinduction method and modified expression plasmid genotypes, improved the expression of soluble TEV protease to 400 mg per litre of cell culture (Blommel & Fox, 2007 ▶). An improvement in the solubility of an MBP-TEV protease fusion over histidine-tagged TEV protease has also been presented by Kapust & Waugh (1999 ▶). Our MBP-fused construct yielded highly soluble 6-His-MBP-3C protease with an average yield of 52 mg (enough to carry out cleavage digestions for 2.6 g recombinant protein) from a 2 l expression volume of bacterial cell culture. Furthermore, this enzyme was stable for at least six months when stored in buffer containing 50% glycerol at 253 K.

Data analysis of enzymatic cleavage reactions and subtractive IMAC reveals the high efficiency of 6-His-MBP-3C protease. Of 208 digestions with 3C protease, including proteins that passed purification and for which concentrations for each fraction in subtractive IMAC were measured, 195 (94%) yielded complete cleavage. Partial cleavage was seen for only 13 of the 208 total digestions performed (6%). However, it should be noted that this was not a consequence of inactive 6-His-MBP-3C protease, as parallel cleavages with different recombinant proteins always cleaved to completion. In each incomplete cleavage the protein was observed to be a multimer based on its apparent molecular weight during SEC. Thus, we hypothesize that 3C cleavage may be incomplete owing to a lack of accessibility of the 3C cleavage site because of oligomeric protein–protein interactions. Another group noted incomplete cleavage of oligomeric proteins and hypothesized that the cleavage tags were not accessible in the oligomers (Kenig *et al.*, 2006 ▶). Of those 195 complete 3C digestions, subtractive IMAC was successful 166 times (85%). A successful IMAC recovers most of the protein in the flowthrough and wash fractions, leaving any contaminants bound to the Ni resin to be removed in the elution fraction. This is analyzed quantitatively by calculating the recovery, or the percentage of total protein (protein obtained after the primary IMAC step in protein purification) recovered after subtractive IMAC, in the flowthrough and wash fractions. The median recovery of input protein in successful sub­tractive IMAC was 80.2%. Non-ideal behavior was seen in 15% of the subtractive IMAC outcomes, in which substantial quantities of cleaved proteins were retained on the second IMAC column and appeared in the elution fraction. If the elution fraction is pooled for further processing, contaminants are reintroduced into the protein sample, including the 3C protease, the His-tag peptide and native IMAC-binding *E. coli* proteins. Depending on the size of the target protein, these impurities may not be separated from the target protein during SEC. Therefore, in almost all cases the elution fraction was not pooled with the flowthrough and wash fractions, and the nonspecific binding of the recombinant protein resulted in lower recovery. Tagless protein may bind to the Ni resin for a variety of reasons, including the presence of surface clusters of histidine residues, metal-binding domains and/or hydrophobic patches that bind to the Sepharose matrix (Bolanos-Garcia & Davies, 2006 ▶). Fortunately, this non-ideal behaviour was observed for only 29 of 195 (15%) successful 3C digestions, lowering the median percentage yield recovered in these 29 instances to 55%. Thus, 3C protease and subtractive IMAC have proven to be reliable for the cleavage and increased purity of polyhistidine-tagged proteins.

**Figure 3:**
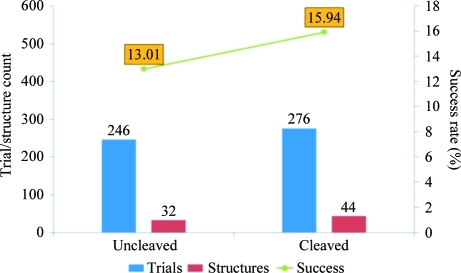
Structure success rate for uncleaved *versus* cleaved proteins. An increase of 2.9% is seen in the structure success rate of cleaved proteins over uncleaved proteins. This is likely to be a consequence of the removal of contaminating Ni-binding *E. coli* proteins.

3C cleavage followed by subtractive IMAC improved the likelihood that a recombinant protein will lead to an X-ray crystallo­graphic structure (Fig. 3 ▶). For this analysis, we included all proteins submitted to the crystallography group that have had sufficient time to undergo crystal trials and yield a structure. 276 proteins that have been cleaved and undergone sub­tractive IMAC led to 44 structures being made available to the scientific community through the Protein Data Bank, a success rate of 15.9%. Alternatively, a total of 246 crystal trials on uncleaved proteins led to 32 structures, a success rate of 13.0%. While this is an increase of only 2.9% in the number of structures solved by the addition of cleavage and subtractive IMAC steps, this seemingly small change represents a significant improvement over the five years of the project. During these five years, the UW-PPG is projected to purify 2000 proteins. If we produce un­cleaved proteins we project a yield of 260 solved structures, but if we cleave and use subtractive IMAC we project a yield of 318 solved structures. Better purity and removal of the histidine tag, which is often disordered, are the most likely contributing factors to the increase of the structure success rate. Owing to this significant increase in success rate, all SSGCID protein purifications performed by UW-PPG include a cleavage step.

Of the structural genomics centers that choose to perform cleavage, 3C protease and TEV protease are most commonly used owing to their high specificity and catalysis of cleavage to completion. Catalytic efficiency is described by the kinetic parameter *k*_cat_/*K*_m_ from Lineweaver–Burk regression analysis, with a higher value indicating a more complete reaction. The literature has shown higher efficiency for 3C protease based on cleavage experiments carried out at 303 K. However, it should be noted that this value is substrate-dependent. Wang *et al.* (1997 ▶) showed that purified 3C protease had a *k*_cat_/*K*_m_ value of 840 *M* ^−1^ s^−1^ for the substrate EALFQ-pNA. Alternatively, kinetics studies by Miladi *et al.* (2011 ▶) showed a much lower *k*_cat_/*K*_m_ (260 *M* ^−1^ s^−1^) for TEV protease. 6-His-TEV protease also had a similar *k*_cat_/*K*_m_ value of 270 *M* ^−1^ s^−1^ for a different substrate and it cleaved only 70% of the fusion protein in an overnight incubation at 303 K when mixed in a 1:14 enzyme:substrate ratio (Fang *et al.*, 2007 ▶). Our ratio of 1:50 enzyme:substrate led to complete cleavage 94% of the time (see below). Therefore, based on the incomplete cleavage by TEV protease and the lower *k*_cat_/*K*_m_ values, 3C protease may be the better option for enzymatic cleavage of recombinant tagged proteins.

The following success rates further attest to the validity and efficiency of the protein-purification protocol with 3C cleavage developed by UW-PPG. Of 315 purification attempts using the outlined protocol, 39 were counted as failed purifications, giving an overall success rate of 87.6%. The average amount of protein delivered for crystallization trials was 53.0 mg and the median preparation was 38.7 mg. This quantity of protein allowed multiple crystal trials and even cocrystallization with multiple ligands in certain cases. Following purification, crystallization trials were set up for each protein according to a rational crystallization approach (Newman *et al.*, 2005 ▶) using the JCSG+ and PACT sparse-matrix screens from Emerald BioSystems (Bainbridge Island, Washington, USA). 0.4 µl protein solution was set up at 289 K with an equal volume of precipitant against an 80 µl reservoir in sitting-drop vapor-diffusion format in 96-­well Compact Jr plates (Emerald BioSystems, Bainbridge Island, Washington, USA). These trials have been completed for 276 cleaved proteins and resulted in the determination of 44 structures, a success rate of 15.9%.

The high success rate further attests that the subtractive IMAC purification method described in this article is highly efficient. However, ongoing process improvements are required for this and other procedures within the SSGCID structure-determination pipeline in order to continuously improve the output and cost-effectiveness of structural genomics. Going forward, UW-PPG plans to focus more attention on target selection prior to purification. Rather than simply picking targets that have passed expression testing, those that show high expression and solubility of the protein product will be prioritized, as these high expressors are 40% more likely to yield a structure than low- or medium-expressing proteins (Choi *et al.*, 2011 ▶). Proteins which have greater expression and solubility going into purification generally have greater purity and yields, which undoubtably contributes to higher structure yields. Also, high solubility in screening means that the protein is not likely to be misfolded and is less likely to aggregate while it is being processed. Therefore, all other considerations being equal, targets with the best expression and solubility profiles during screening will be moved to higher priority for further processing.

Unfortunately, some proteins have low solubility and are prone to becoming insoluble during the purification procedure. If aggregation is observed at any point during a purification, a quick search of PubMed or the Protein Data Bank (PDB) often reveals possible ligands, such as metals, cofactors or substrates, which are added to the proteins. These additives then bind to and theoretically stabilize the protein so that it may continue to be processed. The Structural Genomics Consortium used this approach together with differential scanning fluorimetry (DSF) and differential static light scattering (DSLS) to optimize buffer conditions and screen both generic libraries and focused libraries of ligands, detergents, metals, inhibitors and other additives (Vedadi *et al.*, 2006 ▶). They were able to increase the thermostability of >50% of the 221 proteins tested by varying pH and/or salt concentrations alone. In a more specific example, 84% of 32 kinases that were screened against a library of 500 kinase inhibitors resulted in an increase in thermostability of >4 K upon addition of the identified compound. These statistics provide strong evidence that buffer optimization and ligand addition are legitimate methods for rescuing aggregating proteins. By prioritizing targets with high expression and attempting to stabilize problem proteins, it is the goal of UW-PPG to increase success rates in crystallization trials and to increase the number of structures being deposited in the PDB.

The results presented in this paper demonstrate that the SSGCID protein-production group at the University of Washington has successfully implemented a robust protein-production pipeline that has supported the discovery of over 75 new protein structures a year over the last three years. These structures can be accessed by the scientific community through the PDB and are used in a wide range of other projects, for example structure-based drug design. It is our hope that our efforts may contribute to the expanding knowledge of protein structure and the discovery of new medicines against significant pathogens.
